# Advances in Endoscopic Photoacoustic Imaging

**DOI:** 10.3390/photonics8070281

**Published:** 2021-07-16

**Authors:** Yan Li, Gengxi Lu, Qifa Zhou, Zhongping Chen

**Affiliations:** 1 Beckman Laser Institute, University of California Irvine, Irvine, CA 92617, USA; 2 Roski Eye Institute, Keck School of Medicine, University of Southern California, Los Angeles, CA 90033, USA; 3 The Edwards Lifesciences Center for Cardiovascular Technology, University of California Irvine, Irvine, CA 92617, USA; 4 Department of Biomedical Engineering, University of California Irvine, Irvine, CA 92697, USA

**Keywords:** endoscopic photoacoustic image, ultrasound image

## Abstract

Photoacoustic (PA) imaging is able to provide extremely high molecular contrast while maintaining the superior imaging depth of ultrasound (US) imaging. Conventional microscopic PA imaging has limited access to deeper tissue due to strong light scattering and attenuation. Endoscopic PA technology enables direct delivery of excitation light into the interior of a hollow organ or cavity of the body for functional and molecular PA imaging of target tissue. Various endoscopic PA probes have been developed for different applications, including the intravascular imaging of lipids in atherosclerotic plaque and endoscopic imaging of colon cancer. In this paper, the authors review representative probe configurations and corresponding preclinical applications. In addition, the potential challenges and future directions of endoscopic PA imaging are discussed.

## 1. Introduction

To access comprehensive structural and functional information of internal organs, various imaging technologies, such as optical coherence tomography (OCT), ultrasound (US), and near infrared fluorescence (NIRF) imaging have been developed for endoscopic imaging and used in clinical applications. The main advantage of US and OCT lies in their capability of providing cross-sectional structural information, revealing the underlying layered structure of the biological tissue. US has a large penetration depth and can reveal information that lies in the deeper layers of the tissue. Benefiting from its micron-scale resolution, OCT has the capability to visualize tissue with more details. Near infrared fluorescence and spectroscopy (NIRF and NIRS) are capable of providing molecular contrast with high sensitivity and resolution, but it lacks depth information. Multiphoton can provide high resolution optical sectioning with an imaging depth of several hundreds of micrometers, but it has a limited field of view. Table 1 summarizes the strengths and limitations of these imaging techniques. As shown, no single technique can provide depth resolved structural and functional information simultaneously.

**Figure 1.**
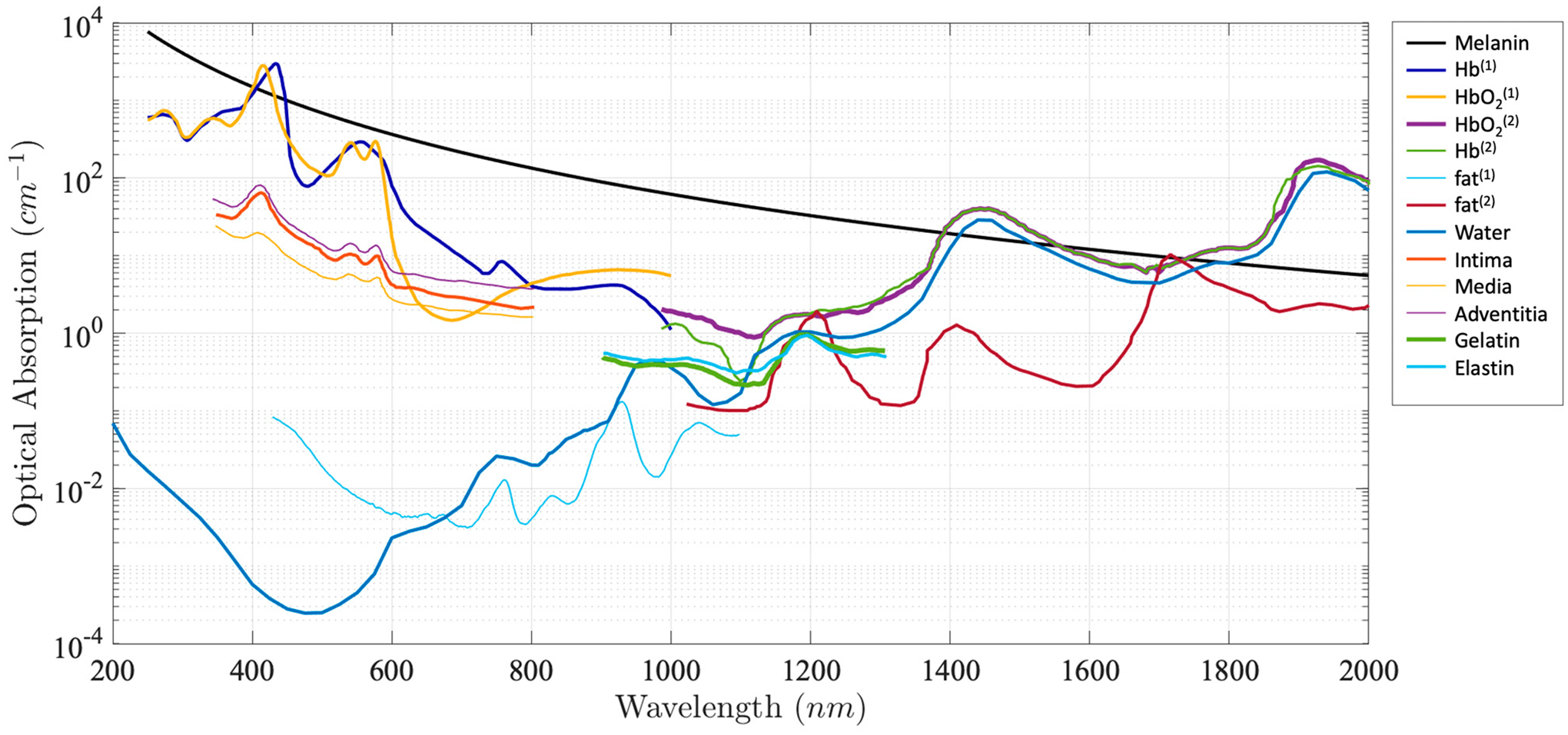
Absorption coefficient spectra of endogenous tissue chromophores [30–33].

Photoacoustic (PA) imaging, an emerging biomedical imaging modality, has the advantage of providing optical absorption contrast with a greater penetration depth compared with conventional optical imaging modalities [4–12]. In PA imaging, the biological tissue is irradiated by a pulsed light. A portion of the optical energy is absorbed by tissue and converted into heat, resulting in a transient pressure rise. This initial pressure acts as an acoustic source that generates an acoustic wave propagating through the tissue. An ultrasonic transducer is often used for detecting the acoustic wave to form PA images. Due to the introduction of a US transducer, US imaging can be incorporated seamlessly. Alternatively, an all optical US sensor, such as a Fabry–Pérot interferometer and microresonator, can be also applied to detect a generated PA signal [13–20]. The intensity of the generated PA signal is proportional to the local absorption coefficient of the tissue, the light intensity, and the Grüneisen parameter, which represents thermoacoustic conversion efficiency. Different biological tissues exhibit different absorption spectra, as shown in Figure 1. Absorption spectra can be used to image and identify a specific tissue chromophore when excited at specific wavelengths that are coincident with the corresponding absorption peaks. For example, 1210 nm and 1720 nm are often used to map lipids in an atherosclerotic lesion [21–26], and 532 nm are often chosen to image vasculature [27–29]. The depth information can be reconstructed by measuring the time of arrival of generated acoustic waves. For endoscopic PA imaging, a miniatured imaging probe is involved, which usually consists of an optical fiber for light delivery and a US sensor for PA signal detection.

As an extension of PA imaging, spectroscopic PA imaging can be performed by applying multiwavelength laser excitation and a spectroscopic analysis for tissue characterization and quantification due to tissue unique spectral signatures. Spectroscopic PA imaging has been investigated in a wide variety of applications. For example, spectroscopic PA has been applied in intravascular imaging to detect and distinguish peri-adventitial and atherosclerotic lipids, one of the main characteristics of vulnerable plaque [7,34,35]. Another important application is to measure blood oxygen saturation (SO_2_), which is a key factor for cancer detection and staging, using the differences in oxyhemoglobin (HbO_2_) and deoxyhemoglobin (HHb) [36,37].

In addition to intrinsic contrast, exogenous optical contrast agents such as gold nanoparticles and indocyanine green (ICG) have been investigated for PA imaging to further enhance the PA signal or provide additional optical contrast. For example, several studies have reported that ICG is able to target lipid loaded macrophages, which can be used in intravascular imaging for vulnerable plaque characterization [38,39]. However, safety will be a main concern if exogenous contrast agents are introduced.

Representative endoscopic PA imaging system and probe design have been reported in recent review articles [40,41]. In this review paper, we will focus on the comparison of different probe designs and further summarize a representative PA imaging system with key parameters of performance.

## 2. Endoscopic Photoacoustic Imaging System

**Figure 2.**
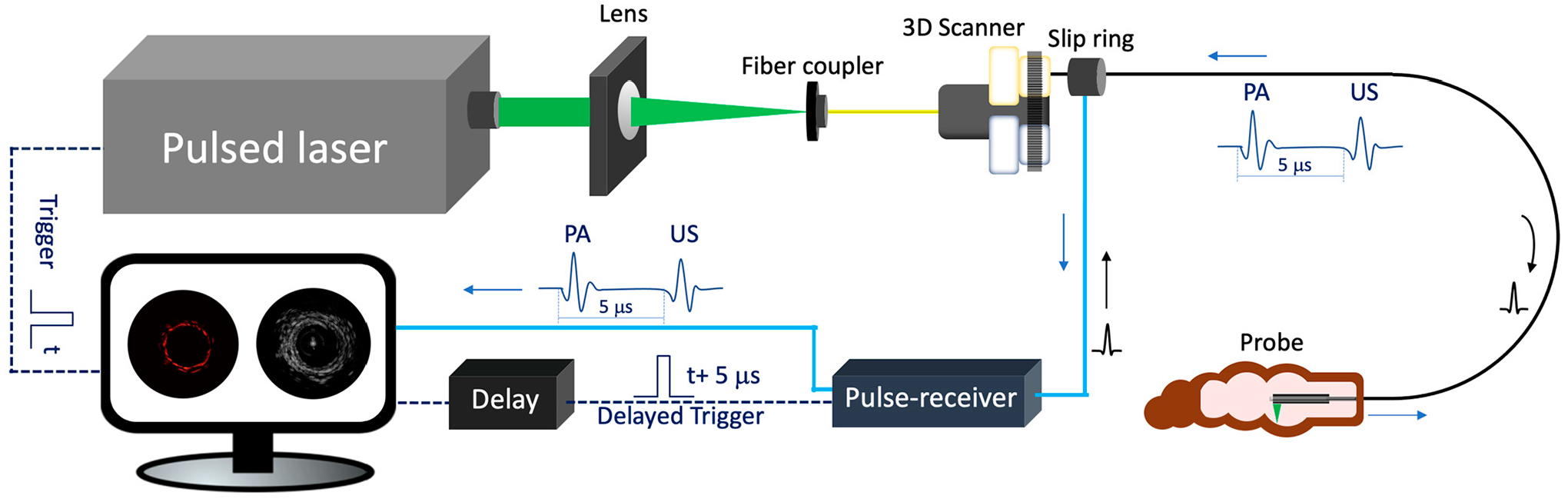
Endoscopic photoacoustic and ultrasound imaging system. PA: photoacoustic. US: ultrasound.

Figure 2 depicts the schematic of a representative endoscopic PA imaging system setup [29]. A nanosecond pulsed laser is used for PA signal excitation. The output laser beam is focused by a condenser lens into the imaging probe to excite the target tissue. A US transducer is used to detect the generated PA signal. Due to the introduction of a US transducer, US imaging can be performed simultaneously. To separate PA and US signals, a delay unit is often applied to delay US pulse emission by a few nanoseconds. For radial scanning, a scanner consisting of an optical rotary joint and slip ring can be utilized to pass optical and electrical signals across rotating interfaces. With this scanning mechanism, the imaging probe can be miniaturized in terms of outer diameter and rigid length. However, nonuniform rotation distortion (NURD) will be a main concern, especially for intravascular imaging. Alternatively, a micromotor can be incorporated into the imaging probe tip to rotate the mirror only, instead of the entire imaging probe. This scanning mechanism will enable uniform rotation at the cost of increased rigid length. Tables 2 and 3 show representative endoscopic PA imaging systems and corresponding key parameters.

A nanosecond laser is one of the key components of a PA imaging system as its repetition rate determines the maximum imaging speed and its energy affects the signal to noise ratio of the PA signal. In most reported studies, a Q-switched Nd: YAG pumped optical parametric oscillator (OPO), Ti: sapphire, or dye laser systems are used for PA signal excitation, in which a tunable wavelength enables spectroscopic PA imaging [14,37,42,47,52,55]. This OPO often operates at a slow repetition rate (~10 Hz) and slow tuning speed. In addition, diode pumped solid state (DPSS) lasers have been introduced into PA imaging due to their high power and repetition rate, so that a high frame rate can be achieved [27,43]. However, DPSS laser based PA systems are incapable of performing spectroscopic PA imaging due to their single wavelength operation.

One of main technical barriers hindering the clinical translation of PA is the shortage of appropriate laser sources that can provide sufficient pulse energy as well as repetition rate. Although the reported fastest imaging speed for in vivo intravascular PA (IVPA) imaging is 25 frames per second, heavy water was applied to enhance the SNR [60]. In addition, Yang et al. adopted two pulsed lasers (operating at wavelengths of 562 nm and 584 nm), instead of an OPO laser, to perform PA imaging of blood vessels for calculating SO_2_ saturation to overcome the slow wavelength sweep speed of the OPO laser. The desired laser should have the following features. (1) High repetition rate while maintaining high pulse energy. (2) High wavelength sweep rate and large sweep range. (3) Compact size for practical use.

## 3. Endoscopic Photoacoustic Imaging System Probe

In order to achieve high quality PA images, an optimal imaging probe that includes an optical fiber to deliver excitation light and a US detector for signal detection is essential. Various probes using different types of light delivery, US detection, and scanning mechanisms have been investigated. Each has its own advantages and limitations. Based on clinical applications, these designs can be categorized into two groups: GI tract and intravascular imaging probes.

### 3.1. GI Tract Endoscope

**Figure 3.**
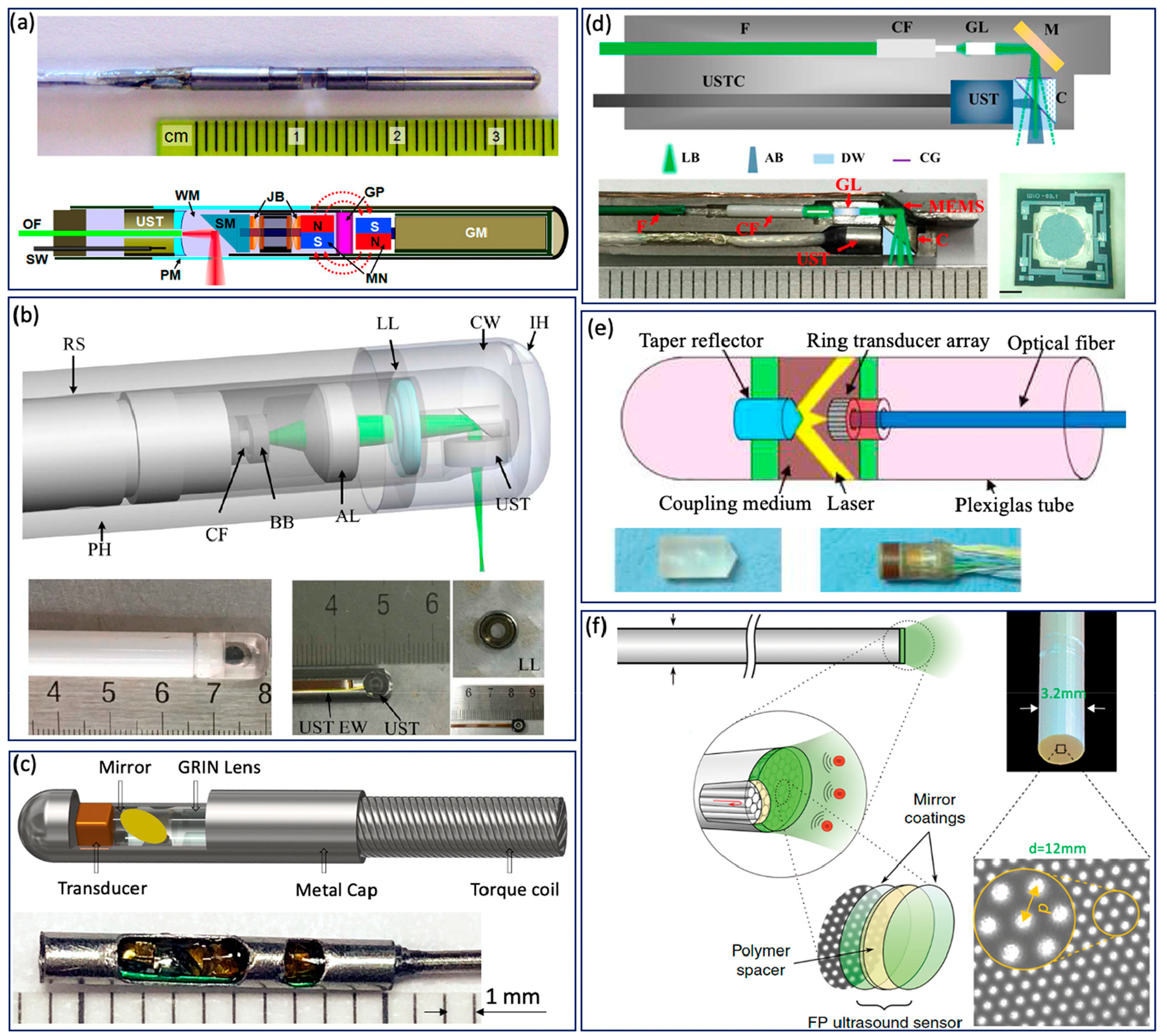
GI Tract PA endoscope. (**a**) Distal scanning based coaxial imaging probe. GM, geared micromotor; GP, glass partition; JB, jewel bearings; MN, magnets; OF, optical fiber; PM, plastic membrane (imaging window); SM, scanning mirror; SW, signal wire; UST, ultrasonic transducer; WM, water medium. Adapted from [42]. (**b**) Proximal scanning based coaxial imaging probe. RS, rotating shaft; LL, liquid lens; CW, coupling window; IH, inject hole; PH, plastic housing; BB, ball bearing; CF, ceramic ferrule; AL, aspheric lens; UST, ultrasonic transducer; UST EW, ultrasonic transducer electric wire. Adapted from [45]. (**c**) Proximal scanning based noncoaxial imaging probe. Adapted from [27]. (**d**) MEMS based imaging probe. F, fiber; CF, ceramic ferrule; GL, GRIN lens; M, MEMS mirror; UST, ultrasound transducer; USTC, ultra- sound transducer cable; C, cube; LB, light beam; AB, acoustic beam; DW, deionized water; CG, cover glass. Scale bar: 0.5 mm [51]. (**e**) Ring-shaped transducer array based imaging probe. Adapted from [50]. (**f**) All optical PA imaging probe. Adapted from [13].

Various GI tract imaging probes have been proposed [6,13,18,27,29,37,42,44,45,47,49–51,61–66], as shown in Figure 3. Yang et al. reported a series of micromotor based endoscopic PA probes [6,37,42,47,63]. The probe consists of a ring-shaped US transducer and an optical fiber which is mounted in the central opening of the ultrasonic transducer. A mirror aligned with the fiber tip and US transducer coaxially is driven by a micromotor to reflect both laser and acoustic beams towards the tissue, as shown in Figure 3a. This imaging probe has a limited field of view due to being blocked by the stainless steel wall. In addition, the imaging speed is relatively slow, because the mirror is rotated through a magnetic coupling mechanism in order to isolate the micromotor from water. To address this issue, Xiong et al. [45] and Li et al. [44] reported several similar coaxial PA imaging probes with different scanning mechanisms, in which the entire imaging probe or probe shell was rotated by an external motor at a frequency of 2 Hz. In addition, the ring-shaped US transducer was aligned with the laser beam coaxially and placed near the imaging window, as shown in Figure 3b. This configuration will avoid extra US signal attenuation caused by the reflection of the mirror, thereby improving sensitivity. To perform high speed and full field of view imaging, Li et al. [27,29] developed a PA probe with torque coil based scanning, in which optical and acoustic components are arranged in a series. Optical and/or acoustic beams are tilted to achieve an overlap between two beams for effective PA signal detection. The imaging speed is up to 50 Hz. The main limitations include limited imaging range and additional steps for achieving coregistered PA and US images due to longitudinal offset between the two beams. In addition to the radial scanning mechanism, Guo et al. [51] and Qu et al. [64] demonstrated a microelectromechanical systems (MEMS) scanning mirror based imaging probe, as shown in Figure 3d, which is able to perform fast raster scanning. The maximum scanning frequency of this MEMS mirror is 500 Hz, which can support a 1000 Hz frame rate with the fast laser. The main limitation is its relatively large probe size due to the use of MEMS. To perform cross sectional imaging without scanning, Yuan et al. [50] developed an imaging probe (Figure 3c) based on a ring-shaped array US transducer, in which a transducer and optical fiber were aligned coaxially. A tapered reflector was used to transform the laser beam into a ring- shaped distribution to illuminate the sample and a 64-element ring transducer array connected with the parallel acquisition system was used to detect the generated PA signal, as shown in Figure 3e. Basij et al. [49] present a phased array US transducer based imaging probe, in which six silica core/cladding optical fibers surrounding the US transducer were used to illuminate the sample. In addition to the traditional US transducer, an optical US sensor (such as a micro ring resonator and FP sensors) has been used in endoscopic PA imaging probes [13,18,65,66]. Figure 3f shows a representative all optical imaging probe, which consists of a fiber bundle and the Fabry–Pérot (FP) US sensor at the fiber tip [13].

### 3.2. Intravascular Imaging Probe

**Figure 4.**
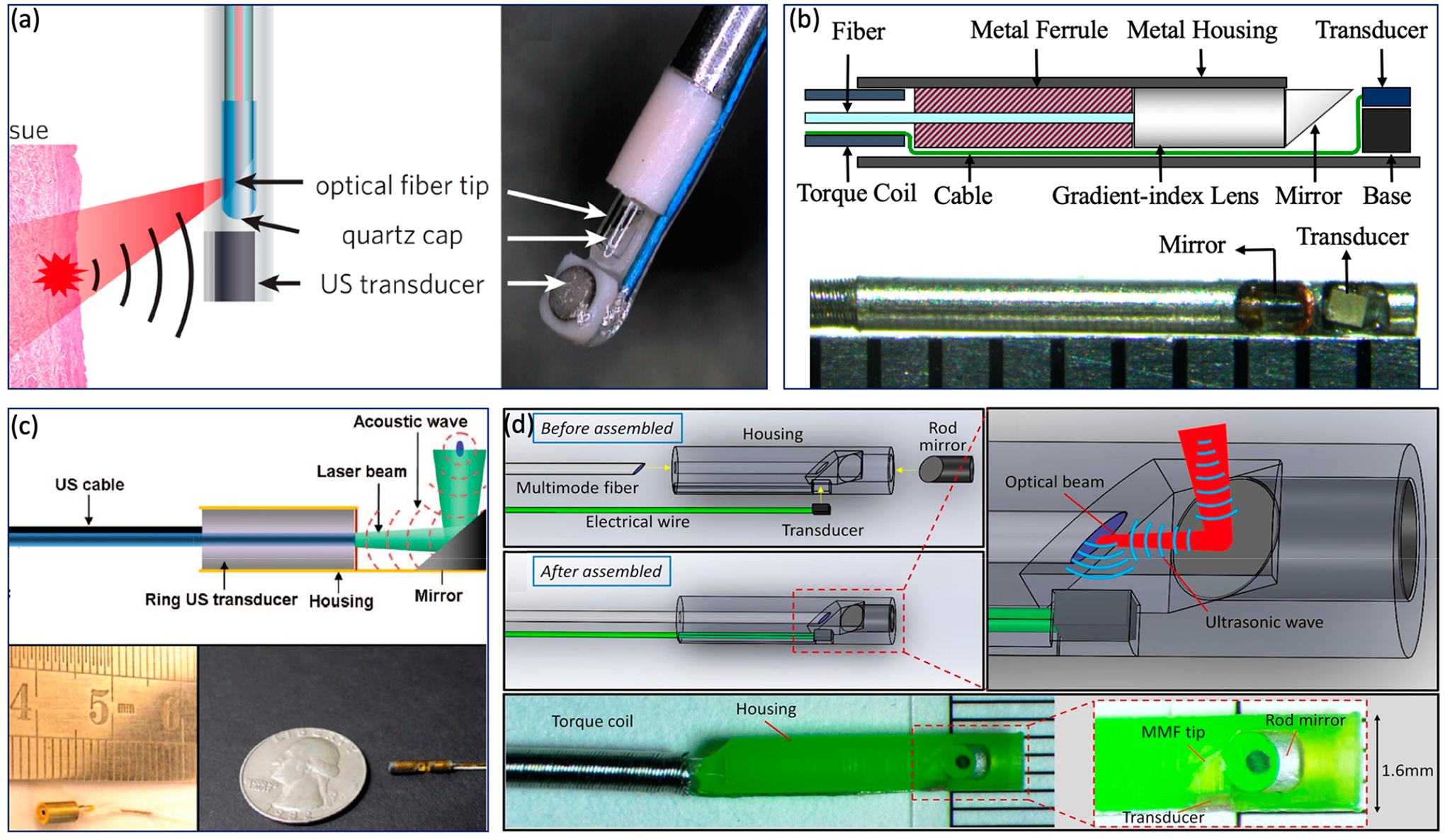
Intravascular imaging probe. (**a**) Sequential alignment and broad illumination. Adapted from [72]. (**b**) Sequential alignment and focusing illumination. Adapted from [22]. (**c**) Coaxial alignment and broad illumination. Adapted from [11]. (**d**) Coaxial alignment and broad illumination. Adapted from [70].

In contrast to the GI tract imaging probe, an intravascular imaging probe needs to be implemented with a smaller diameter and shorter rigid length in order to realize a smooth advance in the tortuous cardiovascular system. Various probe configurations were reported [11,22–25,67–71], as shown in Figure 4. Wu et al. proposed a 0.8 mm noncoaxial probe, in which a multimode fiber end was angle polished to enable total reflection for laser delivery and a side-facing US transducer was aligned in a series for PA signal detection. The lateral resolution of this kind of probe is relatively poor due to the broad illumination [25]. With the same alignment, Li et al. [22] introduced a grin lens at the end of the optical fiber to achieve a quasifocusing illumination (Figure 4b). An improved resolution of ~200 μm was obtained. To further improve lateral resolution, Zhang et al. applied a graded index multimode fiber to replace a multimode fiber for light propagation, which enables a beam self-cleaning effect and offers a lateral resolution of 30 μm [67]. Wang et al. proposed to utilize a tapered fiber (core: from Ø25 to Ø9 μm) instead of a multimode fiber, which contributes to an optimal lateral resolution of 18 μm [68]. However, the above mentioned noncoaxial imaging probes [22,24,25,67–69] have limited imaging range and require additional steps to achieve coregistered PA and US images. To address these issues, Wei et al. [11] and Cao et al. [70] presented coaxial imaging probes, as shown in Figure 4c,d, in which optical and acoustic beams share the same optical path. This configuration can be realized by using a ring-shaped US transducer (Figure 4c) or multireflection scheme (Figure 4d), which enables a long imaging range and automatic image coregistration [11,23,70]. The limitations of these designs include a relatively large size due to the use of the ring-shaped US transducer and degraded sensitivity caused by multiple reflections of the PA/US signal.

### 3.3. Ultrasound Transducer

In addition to improvement in probe configuration, tremendous efforts have been made to develop a miniature US transducer. The piezoelectric material used in an ultrasonic transducer has a significant influence on the performance of the PA/US imaging system. The widely used piezoelectric materials for endoscopic PA imaging include Polyvinylidene fluoride (PVDF) [73], Lithium niobate (LiNbO_3_ or LNO) [37,47,63], lead magnesium niobate-lead titanate (PMN-PT) [26,42], and lead zirconate titanate (PZT) [22,28,70]. The typical acoustic and piezoelectric properties are listed in Table 4.

LiNbO_3_ has the largest Curie temperature (T_c_) among piezoelectric materials. This provides LNO-based transducers with a stable performance in high pulse repetition frequency (PRF) imaging. In addition, the large T_c_ is especially important for PA/US endoscopy because the high intensity laser used in PA could generate lots of heat in the confined endoscopic probe, which could depolarize the piezoelectric materials.

PZT is one of the most conventional piezoelectric ceramics and has been widely used in various applications. PZT for imaging applications usually has electro–mechanical coupling capabilities (k_t_) larger than LNO. By modifying the composition of PZT ceramics, piezoelectric properties can be changed within a very wide range. This provides flexibility in transducer design. For example, to match with most control systems and data acquisition systems, a transducer’s electrical impedance is usually designed to be 50 Ohms. Since PZT’s relative clamped dielectric constant ranges from 580 to 2500, a range of the size and center frequencies can be selected.

PMN-PT is a new class of single-crystal piezoelectric materials with improved piezoelectricity. It has the largest piezoelectric strain constant (d_33_) and k_t_ (larger than 0.58) compared to other common piezoelectric materials [75,77]. Therefore, a PMN-PT based transducer has better SNR, compared to PZT and 1–3 composite PZT transducers [29,78]. In addition, PMN-PT and certain types of PZT are especially appropriate for minimized intravascular transducers (an aperture size smaller than 0.6 mm) due to their high dielectric constant.

PVDF is a soft polymer with unique properties. It has the lowest acoustic impedance compared to other stiff piezoelectric materials. The best acoustic impedance matching to water or biological tissues (~1.5 MRayls) allows US waves to enter the transducer with the least reflection. This compensates its low k_t_ and provides large bandwidth. However, its low dielectric constant highly limits its applications in endoscopy, especially when electrical impedance tuning is unavailable in endoscopic probes.

1–3 composite materials based on bulky piezoelectric materials have been developed for more benefits. Piezoelectric rods in 1–3 composite materials embedded in a low density polymer lower the acoustic impedance and highly enhance the electromechanical coupling coefficient (from 0.58 to 0.94 in the case of PMN-PT), which inherently contributes to better acoustic impedance matching between transducers and water, together with higher sensitivity and improved image resolution [79].

Apart from the above conventional piezoelectric materials and their composites, new materials and technologies are emerging in PA/US endoscopy, including lead free piezoelectric materials [80], micromachine based US transducers (piezoelectric and capacitive micromachined US transducer, PMUT and CMUT) [81–86], and optical based acoustic sensing [87]. They have their own unique advantages, but further developments are still required to prove their practical capabilities in PA/US endoscopy.

### 3.4. Sheath

**Figure 5.**
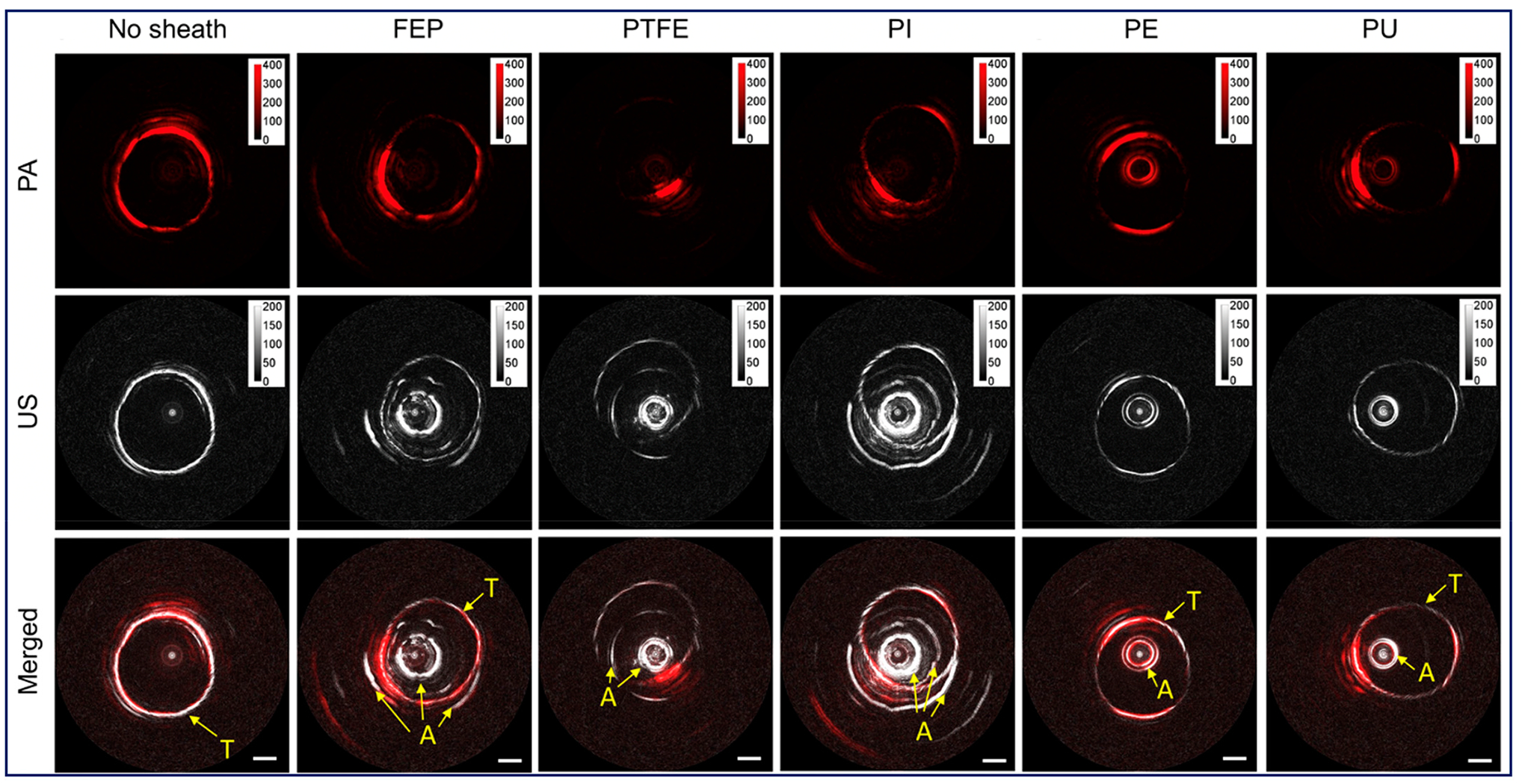
Sheath performance. Adapted from [89]. PE, polyethylene. FEP, fluorinated ethylene propylene. PTFE, polytetrafluoroethylene. PI, polyimide. PU, polyurethane. A, induced artifact from sheath. T, imaging target.

A plastic sheath confines the rotating imaging probe to prevent tissue damage. An ideal sheath for PA imaging should be flexible as well as optically and acoustically transparent to minimize the attenuation of the PA signal and artifacts. Iskander-Rizk et al. investigated the optical and acoustic attenuation of various sheath materials [88], including polyethylene (PE), fluorinated ethylene propylene (FEP), Ethylene tetrafluoroethylene (ETFE), Polytetrafluoroethylene (PTFE), Pebax, OCT sheath, and NIRS sheath. It was found that the NIRS tube exhibits the best performance (optical attenuation: 0.5 dB, acoustic attenuation: 0.3 dB). However, the exact material is proprietary information. The second best is PE, whose attenuation of optical and acoustic signal are 1.5 dB and 1.2 dB, respectively. Cao et al. [89] also tested a series of sheath materials, including FEP, PTFE, polyimide (PI), PE, and polyurethane (PU), to quantify the transmission efficiency of PA and US signals as well as induced PA and US artifacts, as shown in Figure 5. Among them, PEP, PTEF and PI were excluded due to the strong US artifacts. Both PE and PU show better acoustic performance; however, PU exhibits a larger PA transmission efficiency and smaller PA artifacts.

## 4. Application

Endoscopic PA imaging has been investigated in a wide variety of applications, including the characterization of atherosclerosis, GI pathologies, and prostate cancer [9,22–25,37,46,47,54,60,90,91]. This review is mainly focused on cardiovascular and GI tract PA imaging, in which representative applications are discussed.

### 4.1. GI Tract Application

A colonoscopy (i.e., white light endoscopy) is commonly used to evaluate gastrointestinal symptoms. For further diagnosis, physicians also need to excise a small section of suspicious lesions through a colonoscopy for biopsy. While being the standard diagnostic method, a colonoscopy provides only surface morphology of the rectal wall and cannot resolve the layered architecture and functional information. In addition, diagnostic accuracy is limited by sample number and size [92–94]. To address the limitations of a conventional colonoscopy, endoscopic US, OCT, NIRF, and multiphoton have been applied in the GI tract to visualize the layered architecture and microvasculature, detect early stage disease, and assess treatment response with exogenous optical contrast agents, which represents a significant step toward noninvasive comprehensive characterization of GI disease [3,38,95–111]. However, one of the key parameters, SO_2_ saturation, is still lacking. Additionally, contrast agent is necessary during NIRF imaging, which increases invasiveness and may raise safety concerns.

**Figure 6.**
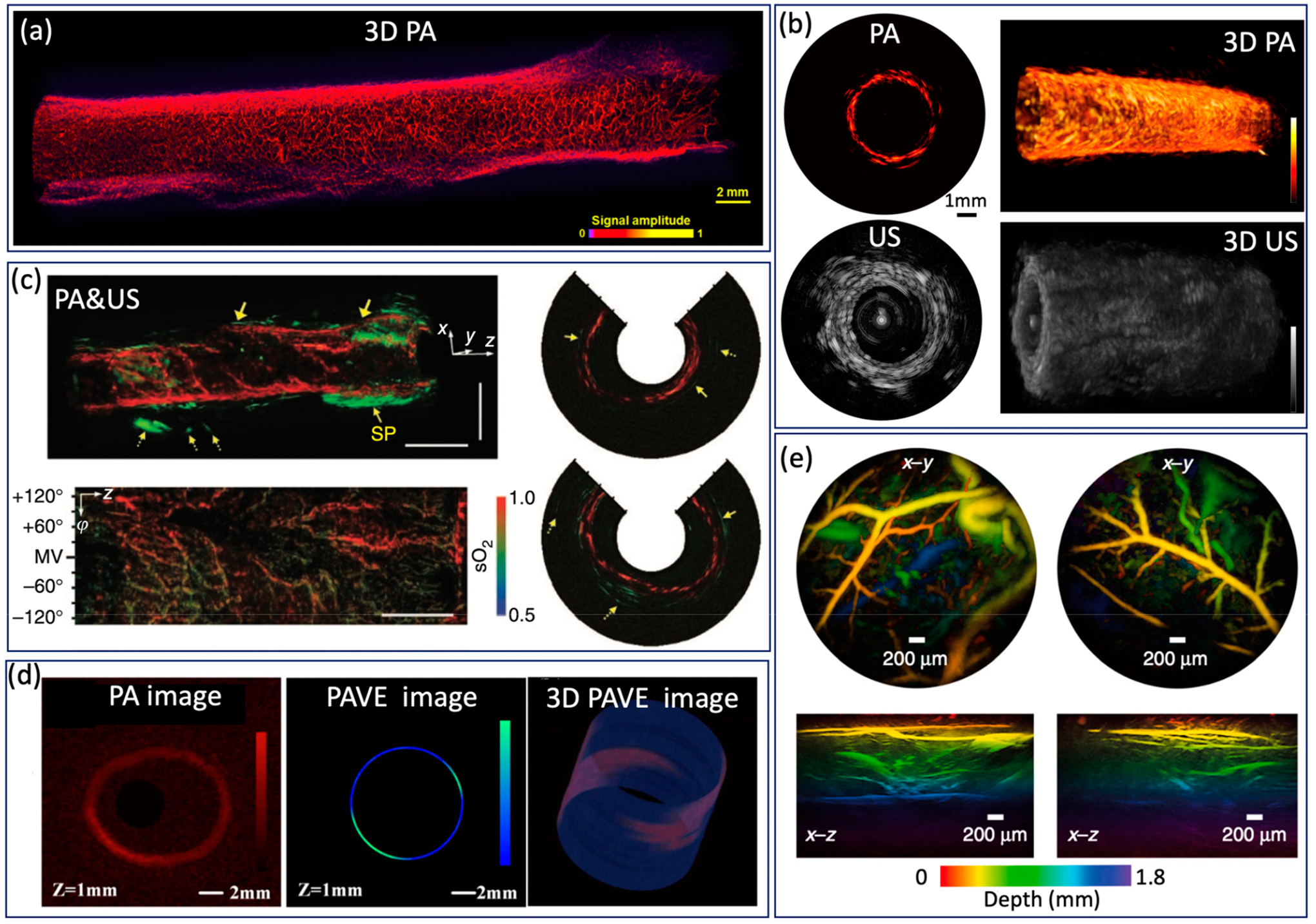
(**a**) Optical resolution PA image from a rat colorectum. Adapted from [47]. (**b**) High speed PA image from a rat rectum. Adapted from [27]. (**c**) SO_2_ levels of a rat colon. Adapted from [37]. (**d**) PA image and PAVE image of a severe reflux esophagitis from a rabbit. Adapted from [46]. (**e**) PA image of mouse abdominal skin microvasculature. Adapted from [13].

Endoscopic PA imaging provides molecular contrast with depth information, which allows for the simultaneous visualization of structural and functional information. It has attracted intensive research interest and been applied in GI tract for characterization of diseases in GI tract by mapping vasculature, measuring SO_2_ saturation, and evaluating elasticity. Yang et al. [47] present a high-resolution endoscopic PA system, which enables visualization of vasculature with a much finer resolution of 10 μm and an imaging speed of 2 Hz, as shown in Figure 6a. However, imaging speed is main limitation for clinical application. To achieve real time imaging, Li et al. [27] developed a high speed PA imaging system, which is able to perform PA and US imaging simultaneously with an imaging speed up to 50 Hz. Nevertheless, the lateral resolution is about ~300 μm, which makes it difficult to identify microvascular systems clearly. To obtain functional information about the GI tract, Yang et al. [37] demonstrated a functional PA and US system by applying an excitation of multiple wavelengths, which allows the simultaneous visualization of vasculature, SO_2_ level, and the morphology of a rat colon, as shown in Figure 6c. This is the first demonstration of measuring SO_2_ in the GI tract and represents a significant step toward comprehensive diagnosis, but the imaging speed is still a main concern for clinical translation of this technology. Jin et al. further extend PA imaging to perform elastography using phase sensitive PA imaging [46], in which tissue biomechanics and morphology can be obtained by detecting the PA phase and PA amplitude information, respectively. Ex vivo experiments were performed to demonstrate the feasibility of the proposed system, as shown in Figure 6d. The accuracy and speed of imaging still need to be further improved for in vivo application. To realize noncontact endoscopic imaging, Ansari et al. demonstrated an all optical endoscopic imaging probe with a Fabry–Pérot (FP) polymer-film as the US sensor [13], which can provide high resolution 3D vasculature of mouse abdominal skin. This approach avoids the need for separate and bulky detectors, providing more flexibility. However, the shallow penetration depth (~2 mm) and long acquisition time (25 min for two images) inhibit clinical application.

### 4.2. Intravascular Application

**Figure 7.**
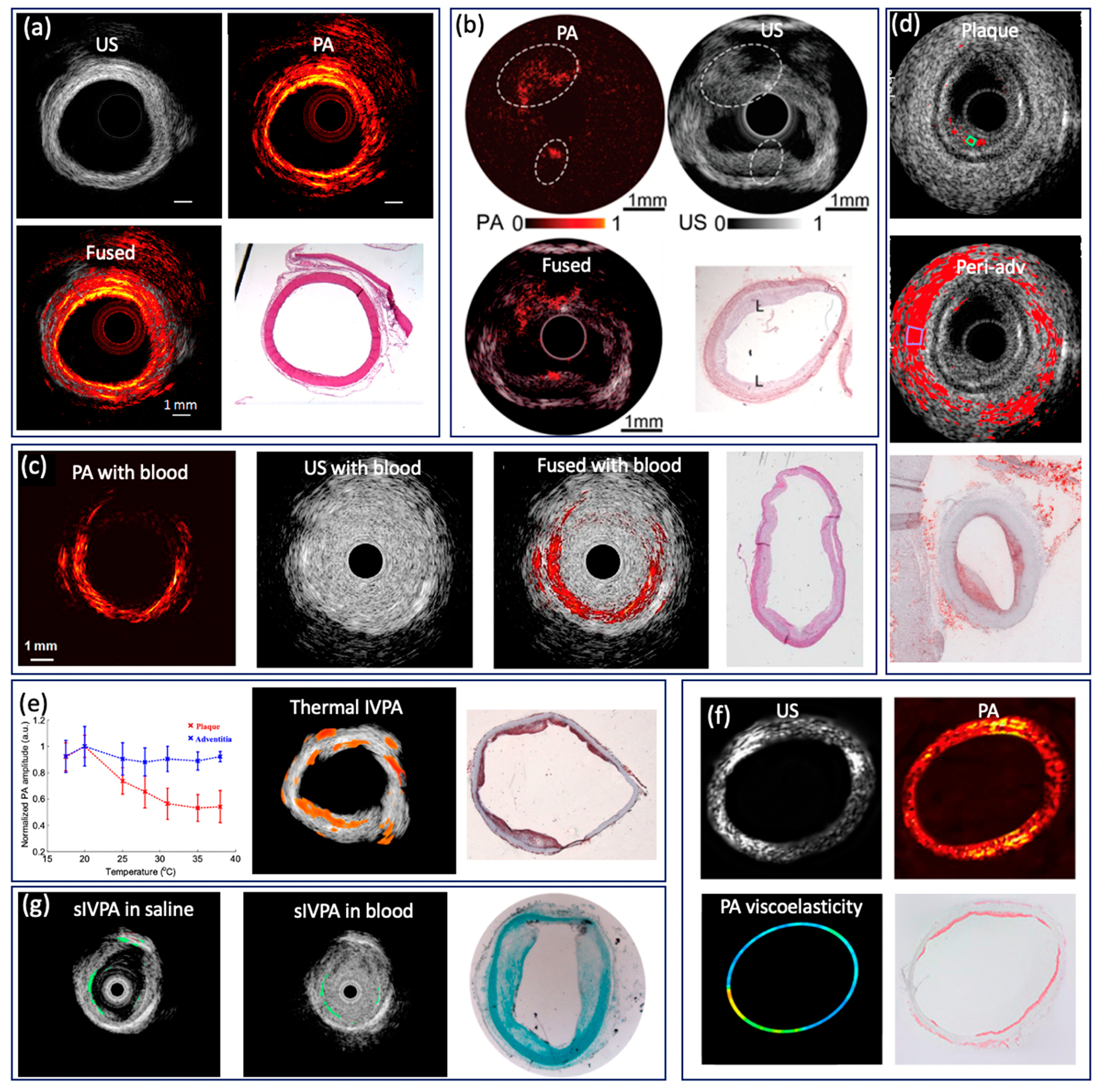
(**a**) PA/US image of rabbit aorta based on 532 nm pulsed laser. Adapted from [53]. (**b**) PA/US image of rabbit aorta based 1725 nm pulsed laser. Adapted from [24]. (**c**) IVPA/IVUS imaging of atherosclerotic rabbit aorta in the human RBCs solution. Adapted from [91]. (**d**) Atherosclerotic and peri-adventitial lipids map from human coronary artery based on wavelength correlation. Adapted from [35]. (**e**) Thermal IVPA images of atherosclerotic rabbit aorta. Adapted from [132]. (**f**) PA, US, elasticity images of rabbit aorta. Adapted from [56]. (**g**) Spectroscopic IVPA image of gold nanorods. Adapted from [133].

In clinical practice, computed tomographic angiography is routinely performed to identify the stenotic region caused by plaque formation via visualization of coronary arteries in two dimensions; however, it lacks the spatial resolution necessary to resolve tissue-level information of the arterial wall, hence the inadequacy in studying vulnerable plaques [112–114]. The development of modern intravascular imaging techniques aims to address this limitation in identifying vulnerable plaques. Intervascular US (IVUS) and OCT (IVOCT) are currently the most significant clinical adaptations. The large penetration depth of IVUS enables full depth visualization of the coronary lumen, blood vessel wall, and atherosclerotic plaque formation, and therefore has been routinely utilized in clinical practices since the early 2000s [115–118]. IVOCT, which has high resolution of 1–15 μm, has the capability to measure fibrous cap thickness [119,120]. Nevertheless, IVOCT suffers from a shallow penetration depth and has limited utility in accessing larger plaques, and IVUS lacks the resolution for visualizing microstructure. In addition, both IVUS and IVOCT have limited sensitivity for studying chemical composition and quantifying tissue mechanical properties, which are key indicators of plaque vulnerability [121–124]. To obtain the chemical composition of a plaque, intravascular NIRF and NIRS capable of providing molecular contrast with high sensitivity have been applied to characterize the intralesion lipid content, but these techniques lack depth information [125–129]. Conversely, intravascular PA (IVPA) imaging is able to provide extremely high molecular contrast while maintaining the superior imaging depth of US imaging [50,130,131], which can map blood vessel wall and lipid content simultaneously. Intravascular imaging of atherosclerotic specimens from cadaver and animal models has been demonstrated [9,11]. Li et al. reported a 532 nm intravascular imaging system and imaged a normal rabbit aorta [53]. Figure 7a shows US, PA, fused images, and an H&E histology photo, in which different layers (intima, media, and adventitia) of a vessel wall can be identified. To map the lipid component in plaque, several research teams investigated IVPA imaging with excitation wavelengths of 1210 and 1720 nm [9,22–25,54,60,91]. According to the absorption spectrum of lipids, 1720 nm is considered the optimal wavelength for IVPA imaging because of the higher absorption coefficient and longer penetration depth. Piao et al. reported an intravascular PA imaging system based on a 1.7 micron pulsed laser and imaged an atherosclerotic rabbit aorta, as shown in Figure 7b [24]. In the image, PA signal was observed at the upper left and lower region (encircled), which indicates the presence of lipid. In addition, the intimal thickening can be found in the corresponding locations (encircled) in the IVUS image, which agrees well with the PA image. The advantage of the 1720 nm band for the IVPA imaging of lipids was also confirmed by Wang et al., who reported a threefold enhancement of the IVPA signal from excitation at 1720 nm vs. excitation at 1210 nm, and demonstrated that IVPA has the capability of imaging lipids through luminal blood using the 1720 nm wavelength [91], as shown in Figure 7c. Considering that lipid within atherosclerotic plaques is the key determining factor, Jansen et al. reported a spectroscopic IVPA imaging system to identify and distinguish atherosclerotic lipid and peri-adventitial lipid. Based on a cholesterol PA spectrum, as a reference for atherosclerotic lipids, and a peri-adventitial reference spectrum, atherosclerotic and peri-adventitial lipids can be well distinguished using correlation with six wavelengths, as shown in Figure 7d. With three well chosen wavelengths, a satisfactory distinction can be achieved. However, with only two wavelengths, atherosclerotic and peri-adventitial lipids can be identified but not distinguished. Additionally, Wang et al. reported a thermal IVPA imaging system, which is able to distinguish lipid types using different temperature dependent PA responses from lipid in plaques and lipid in periadventitial tissue [132], as shown in Figure 7e, where atherosclerotic lipid was mapped overlaid on a US image. In addition to visualizing tissue composition, Wang et al. extended IVPA to quantify vessel elasticity through the PA phase, where a smaller elastic modulus can be found in the plaque region, as shown in Figure 7f [56]. Elasticity of vessel wall is one of the key indicators, as the stress in a fibrous cap is altered by its thickness and macrophage infiltration, which provides additional information for comprehensive characterization of plaque. Furthermore, Yeager et al. investigated IVPA imaging of exogenously labeled atherosclerotic plaque through luminal blood using gold nanorods conjugated with PEG [133]. The nanoparticle is able to label sites of atherosclerosis as well as microphases, as shown in Figure 7g. The addition of a exogenous contrast agent can further improve sensitivity, and make imaging through blood possible. Currently, the main limitation of the above mentioned IVPA system is the slow imaging speed caused by the slow repetition rate of the pulsed laser. In 2017, Wu et al. improved the imaging speed by adopting a 5 kHz OPO laser, but they had to apply heavy water to enhance the SNR due to the small pulse energy [25]. A pulsed laser that can provide sufficient pulse energy as well as a fast repetition rate is necessary for the clinical translation of IVPA.

## 5. Discussion

Endoscopic PA imaging provides functional and molecular information, thereby supplementing conventional endoscope to obtain more comprehensive information. The feasibility has been demonstrated in cadaver tissue and animal model. Although rapid progress has been made, most of the work is still in its preclinical stage. More efforts need to be made to further facilitate clinical translation.

PA signal detection plays an important role in achieving high quality PA images. Although better detection efficiency can be achieved using an imaging probe with coaxial configuration, the probe size will be increased due to the introduction of the ring-shaped transducer. To address this issue, a transparent US transducer could be a possible solution and allow for coaxial alignment without increasing probe outer diameter. LiNbO_3_ crystals are commonly used in transparent US transducers, and applications for these transducers in microscopic and endoscopic PA imaging have been demonstrated by several research groups [134–136]. However, the sensitivity of LiNbO_3_ based US transducers are limited due to their relatively low piezoelectric coefficient and electromechanical coupling factor. In 2020, Qiu et al. [137] fabricated a transparent PMN-PT crystal using an alternating-current electric field. These crystals exhibit near perfect transparency, an ultrahigh piezoelectric coefficient, an excellent electromechanical coupling factor, and a large electro-optical coefficient. Dangi et al. [138] applied this transparent PMN-PT based US transducer in PA imaging. This high sensitivity transparent US transducer could provide more flexibility of probe design, further facilitating the translation of this endoscopic PA imaging technology to clinical use. Furthermore, FP US transducers show great potential in endoscopic PA imaging. In addition to more design flexibility, FB US transducers are free of electromagnetic interference and perform noncontact imaging, which allows for more applications, such as airway PA imaging.

In addition to molecular contrast, functional information is essential for the characterization of target tissue. Since the first demonstration of PA imaging in biomedical imaging in the mid 1990s, several functional extensions of PA imaging have been investigated, including spectroscopic imaging, elasticity, Doppler flowmetry [139,140], and thermometry. Both spectroscopic PA imaging and PA elastography have been applied in endoscopic imaging to identify and quantify specific tissue chromophores as well as elasticity evaluation. PA Doppler flowmetry, based on the Doppler principle, was proposed to measure blood flow velocity and validated in a phantom experiment. Introducing PA Doppler flowmetry in endoscopic PA imaging would be useful because blood velocity is a key physiology parameter of tissue metabolism, which can be used to characterize GI tumors as well as monitoring therapeutic responses. Furthermore, as the PA signal is proportional to the Grüneisen coefficient, that is, temperature dependence, PA imaging has been investigated as a way to map temperature distribution in tissue [141–143]. The addition of PA thermometry in endoscopic imaging will allow for the real time, noninvasive temperature monitoring of energy based treatments.

Integrating PA imaging with other contemporary imaging modalities would provide more comprehensive assessments; this benefit will speed up clinical translation. Dai et al. [144] and Leng et al. [145] reported a trimodality intravascular imaging system which combined PA, US, and OCT for the characterization of atherosclerosis. The addition of OCT contributes to the identification of thin fibrous caps. Furthermore, Zhang et al. [146] developed a combined IVPA and autofluorescence imaging system, which is able to detect lipid core and intraplaque hemorrhage simultaneously. Optical coherence elastography (OCE) can resolve a localized displacement in subnanometers and is therefore ideal for studying the elasticity of biological tissue, which can be incorporated into PA imaging to enable simultaneous visualization of morphology, molecular contrast, and biomechanical properties to further improve diagnostic outcome [121,147,148].

With continuous advancement, PA will be a powerful tool that provides a quantitative means to benchmark and evaluate therapies. Subsurface architecture, vasculature morphology, and SO_2_ saturation are key parameters for the evaluation of GI disease, so endoscopic PA can be used for early detection and staging of GI cancer, as well as monitoring and guiding surgery (such as the identification of tumour margins). With exogenous optical contrast agents, endoscopic PA allows for real time monitoring of the therapeutic response of drugs. For intravascular imaging, the addition of molecular information and elasticity would significantly improve the accuracy of identifying vulnerable plaque.

## 6. Conclusions

In summary, endoscopic PA imaging provides noninvasive techniques for quantitative and dynamic evaluation of tissue physiology and pathophysiology in vivo. With different probe configurations and functional extensions, endoscopic PA imaging will find a broad range of clinical applications.

## Figures and Tables

**Table 1.** Performance comparison of endoscopic imaging modalities.

	Resolutuon	Molecular Contrast	Imaging Depth	Main Limitation
OCT [1]	Axial: ~10 μm Lateral: ~30 μm	N	1–2 mm	Shallow penetration No molecular contrast
US [2]	Axial: ~100 μm Lateral: ~300 μm	N	<6 cm	Low resolution No molecular contrast
NIRF/NIRS	Lateral: ~10 μm	Y	Surface	No depth information
Multiphoton [3]	Axial: 12 μm Lateral: 0.8 μm	Y	~300 μm	Limited field of view Slow imaging speed
PA	Axial: ~100 μm Lateral: ~20–300 μm	Y	<6 cm	-

**Table 2.** Representative endoscopic photoacoustic imaging systems for GI tract.

Study	Laser	US Sensor	Coaxial	Dimension (mm)	Frame Rate	PA Resolution	Scanning Mechanism	Application	Functional Imaging
Yang et al. [42]	Tunable dye laser 584 nm	F: 4 mm, *f*_0_: 33 M Ring-shaped PMN-PT	Y	OD: 2.5 mm RL: 35 mm	4 Hz	L: 100 μm A: 58 μm	Micromotor	In vivo rat colon	-
He et al. [43]	DPSS laser: 2 kHz, 532 nm	Focus: 7 mm, *f*_0_: 30 M Ring-shaped PVDF	Y	OD: 18.6 mm RL: 20 mm	2.5 Hz	L: 80 μm A: 55	Torque coil-based scanning	Ex vivo pig esophagus	-
Li et al. [44]	8 kHz, 527 nm	Unfocused, *f*_0_: 15 MHz	Y	OD: 8 mm Rigid	2 Hz	L: 40 μm A: 125	Shaft based scanning	In vivo rabbit rectum	-
Xiong et al. [45]	10 kHz, 527 nm	Unfocused, *f*_0_: 15 MHz	Y	OD: 9 mm Rigid	-	L: 91 μm A: 121	Shaft	In vivo rabbit rectum	-
Jin et al. [46]	100 kHz to 5 MHz, 1064 nm	Unfocused, *f*_0_: 6 MHz	N	OD: 1.2 mm	-	L: 37 μm A: 253 μm	Shaft	In-situ esophageal tumor	viscoelasticity
Yang et al. [47]	Q-switched diode-pumped Nd:YAG laser, 8 kHz, 532 nm	F = 4.4 mm, *f*_0_: 42 MHz, Ring-shaped LiNbO_3_	Y	OD: 3.8 mm	2 Hz	L: 10 μm A: 50 μm	Micromotor	In vivo rat colorectum	-
Liu et al. [48]	Q-switched lasers, 10 kHz, 532 nm	F = 17 mm, *f*_0_: 15 MHz, Ring-shaped PVDF	Y	OD: 12 mm Rigid	5 Hz	L: 40 μm A: 60 μm	Micromotor	In vivo rabbit rectum	-
Yang et al. [37]	Tunable dye laser 562 nm, 584 nm	F = 5.2 mm, *f*_0_: 36 MHz, Ring-shaped LiNbO_3_	Y	OD: 3.8 mm R: 38 mm	4 Hz	L: 80 μm A: 55 μm	SO_2_ level	In vivo rat colon	SO_2_ level
Basij et al. [49]	Nd:YAG/OPO laser 532 nm	64-element phased-array, *f*_0_: 5–10 MHz	N	OD: 7.5 mm	-	L: 378 μm A: 308	Phase array ultrasound	Phantom	-
Yuan et al. [50]	Nd:YAG laser, 20 Hz, 1064 nm	64-element ring-shaped array, *f*_0_: 6 MHz,	N	OD: 30 mm Rigid	-	L: 2.4 mm A: 320 μm	transducer array	Ex vivo pig Colorectal tissue	-
Guo et al. [51]	Nd:YAG laser, 20 kHz, 532 nm	Unfocused, *f*_0_: 10 MHz	N	OD: 6 mm Rigid	1/8 Hz	L: 10.6 μm A: 105	MEMS scanning	Ex vivo Mouse colon tissue	-
Ansari et al. [13]	Nd:YAG laser, 20 Hz, 1064 nm	Fabry-Pérot (FP) polymer-film	Y	OD: 3.2 mm	25 mins/volume	L: 45 μm A: 31 μm	galvanometer	In vivo mouse skin	-
Li et al. [27]	DPSS laser, Single Shot to 300 kHz 532 nm	Unfocused, *f*_0_: 45 MHz PMN-PT	N	OD: 1.5 mm A: 11 mm	50 Hz	L: 250 μm A: 50 μm	Torque coil-based scanning	In vivo rat rectum	-

**Table 3.** Representative intravascular photoacoustic imaging systems.

Study	Laser	US Sensor	Coaxial	Dimension (mm)	Frame Rate	PA Resolution	Scanning Mechanism	Application	Functional Imaging
Ji et al. [52]	OPO laser, 10 Hz, 750 nm	dual element unfocused transducer *f*_0_: 19 MHz	N	OD: 1.2 mm R: 20 mm	-	L: 13 μm A: 127 μm	Torque, coil based scanning	Ex vivo rabbit aorta	-
Li et al. [53]	Q-switched Nd:YAG laser, 10 Hz, 532 nm	Dual element unfocused transducer *f*_0_: 35 and 80 MHz	N	OD: 1.2 mm	-	L: 232/181 μm A: 59/35 μm	Torque, coil based scanning	Ex vivo rabbit aorta	
Piao et al. [24]	OPO laser, 500 Hz, 1725 nm	Unfocused, *f*_0_: 45 MHz	N	OD: 1 mm R: 6 mm	1 Hz	L: 350 μm A: 60 μm	Torque, coil based scanning	Ex vivo rabbit aorta	
Jansen et al. [34]	OPO laser, 10 Hz, 1125:2:1275	Unfocused, *f*_0_: 44.5 MHz, PMN-PT	N	OD: 1 mm	-	-	Torque, coil based scanning	human atherosclerotic coronary artery, ex vivo	Spectroscopic imaging
Wang et al. [54]	OPO laser, 10 Hz, 1720 nm	Unfocused, *f*_0_: 40 MHz	N	OD: 2.2 mm	-	-	Torque, coil based scanning	In vivo rabbit aorta with blood	-
Mathews et al. [14]	Tunable dye laser, 565 to 605 nm	Fabry–Pérot (FP) polymer film	N	OD: 1.25 mm	1/15 Hz	L: 18 μm A: 45 μm	Torque, coil based scanning	Phantom	-
Zhang et al. [55]	OPO laser, 10 Hz, 720, 760 nm	Unfocused, *f*_0_: 20 MHz	N	OD: 1.8 mm	-	L: 380 μm A: 100	Torque, coil based scanning	In vivo rabbit aorta with blood	Spectroscopic imaging
Wang et al. [56]	OPO laser, 10 Hz, 1700 nm	Unfocused, *f*_0_: 15 MHz	N	OD: 1.1 mm	-	L: 94 μm A: 122 μm	Torque, coil based scanning	Ex vivo rabbit aorta	Elasticity imaging
Wei et al. [11]	Q-switched Nd:YAG laser, 10 Hz, 532 nm	Unfocused, *f*_0_: 39 MHz Ring-shaped	Y	OD: 2.3 mm	-	L: 230 μm A: 34 μm	Rotating target	Ex vivo rabbit aorta	-
Xie et al. [57]	8 kHz to 100 kHz, 1064 nm	Unfocused, *f*_0_: 40 MHz PZT	N	OD: 0.9 mm	100 Hz	-	Torque, coil based scanning	In vivo rabbit aorta with nanoparticles	-
Hui et al. [21]	KTP-based OPO, 500 Hz, 1724 nm	F: 3 mm, *f*_0_: 35 MHz Ring-shaped	Y	OD: >2.5 mm	1 Hz	L: 260 μm A: 102 μm	Torque, coil based scanning	Ex vivo human femoral artery	-
Wu et al. [25]	Periodically-poled LiNbO_3_ OPO, 5 kHz 1720 nm	Unfocused, *f*_0_: 40 MHz	N	OD: 1.3 mm with sheath	20 Hz	-	Torque, coil based scanning	In vivo swine coronary lipid model	-
Lei et al. [58]	OPO laser, 2.5 kHz, 1720 nm	Unfocused, *f*_0_: 50 MHz	N	0.7 mm	5 Hz	L: 209 μm A: 61 μm	Torque, coil based scanning	Ex vivo thoracic aorta mouse	-
Bai et al. [59]	OPO laser, 10 Hz, 1210 nm	Unfocused, *f*_0_: 40 MHz PZT	N	1.1 mm	1/160 Hz	L: 19.6 μm A: 38.1 μm	Torque, coil based scanning	Ex vivo phantom	-
Li et al. [22]	OPO laser, 1 kHz 1210 nm, 1720 nm	Unfocused, *f*_0_: 40 MHz PZT	N	0.9 mm	5 Hz	L: 200 μm A: 100 μm	Torque, coil based scanning	Ex vivo phantom	Spectroscopic imaging

**Table 4.** The acoustic and piezoelectric properties of common piezoelectric materials. K_S_: clamped relative dielectric constant. Z: acoustic impendence. d_33_: piezoelectric constant. K_t_: electro–mechanical coupling coefficient. T_C_: Curie temperature.

Materials	K_x_	Z (MRayl)	d_33_ (pC/N)	k_t_	T_c_ (°C)	Bandwidth
LiNbO_3_ [6,37,47,63,74]	39	34.1	6.2	0.49	1210	60–70%
PMN-PT [26,42,75]	797	36.9	1430–2500	0.58–0.62	130–140	60–80%
PZT [22,28,70]	850–2500	22–23	500–600	0.51–0.53	150–360	60%
PVDF [73,76]	6	3.9–4.1	20–30	0.15–0.2		83%
